# Single-Molecule Fluorescence Methods to Study Plant Hormone Signal Transduction Pathways

**DOI:** 10.3389/fpls.2017.01888

**Published:** 2017-11-02

**Authors:** Song Song, Jian Chang, Chongjun Ma, Yan-Wen Tan

**Affiliations:** State Key Laboratory of Surface Physics, Department of Physics, Fudan University, Shanghai, China

**Keywords:** plant signaling pathway, plant hormone, intrinsic molecular mechanism, *in vitro*, single-molecule fluorescence

## Abstract

Plant-hormone-initiated signaling pathways are extremely vital for plant growth, differentiation, development, and adaptation to environmental stresses. Hormonal perception by receptors induces downstream signal transduction mechanisms that lead to plant responses. However, conventional techniques—such as genetics, biochemistry, and physiology methods—that are applied to elucidate these signaling pathways can only provide qualitative or ensemble-averaged quantitative results, and the intrinsic molecular mechanisms remain unclear. The present study developed novel methodologies based on *in vitro* single-molecule fluorescence assays to elucidate the complete and detailed mechanisms of plant hormone signal transduction pathways. The proposed methods are based on multicolor total internal reflection fluorescence microscopy and a flow cell model for gas environment control. The methods validate the effectiveness of single-molecule approaches for the extraction of abundant information, including oligomerization, specific gas dependence, and the interaction kinetics of different components.

## Introduction

Plant hormones are naturally occurring organic substances that influence physiological processes at low concentrations (Davies, 1995). In addition to the five classical phytohormones, namely auxins, abscisic acid (ABA), cytokinin (CK), gibberellins (GAs), and ethylene, additional compounds have been recognized as hormones, including brassinosteroids (BRs), jasmonate, salicylic acid, nitric oxide, and strigolactones (Santner and Estelle, 2009). These hormones govern every aspect of plant biological processes, including developmental processes, signaling networks, and responses to biotic and abiotic stresses (Bari and Jones, 2009). Auxins regulate diverse processes, such as cell enlargement, cell division, tropic responses, root initiation, and vascular tissue differentiation (Woodward and Bartel, 2005). ABA modulates stomatal closure, inhibits shoot growth, and induces storage protein synthesis in seeds (Davies, 2010). GAs regulate stem growth, fruit setting, and fruit growth and induce seed germination (Davies, 2010). Different hormones regulate distinct members of protein families; however, they may also share components and modulate similar processes (Nemhauser et al., 2006).

The past decades have witnessed the remarkable progress of plant hormone research. Molecular models of plant hormone action, including receptors and their downstream signal transduction components, have been identified and studied using genetic, analytical, biochemical, and physiological approaches. The complete sequencing of the *Arabidopsis* genome, the systematic identification of knockout mutations in CK response, and genetic crosses have revealed the functions of the CK family (Ferreira and Kieber, 2005). Mass spectrometry combined with liquid chromatography (LC-MS) or gas chromatography (GC-MS) can be used for the separation, quantification, and analysis of small compounds such as ABA (Müller and Munnébosch, 2011), auxins (Barkawi et al., 2010), and BRs (Shigeta et al., 2011). Plant hormones are usually transduced by protein–protein interactions. NRT1/PTR FAMILY proteins are the transporters of nitrate, ABA, GAs, and auxins. Modified yeast two-hybrid (Y2H) systems with NRT1/PTR FAMILY have been developed to investigate GA and ABA transport activities (Chiba et al., 2015). By using a pull-down assay coupled with immunoaffinity chromatography, a direct functional association between ABA signaling and RNA processing was established through the interactions of ABA, FCA, and FY with 3′-end RNA processing machinery (Razem et al., 2006). Isothermal titration calorimetry quantified the affinity and kinetics of peptide hormone iminodiacetate and its signaling compounds (Santiago et al., 2016).

Plant hormone signaling pathways are complicated. Although GC- and LC-MS are the preferred methods for the quantitative analysis of plant hormones, they are unsuitable for unstable and highly polar molecules (Metz et al., 2007). Coimmunoprecipitation assay and Western blotting are usually used to analyze moderately stable and strong protein–protein interactions (Persani et al., 2007). However, specific antibodies or protein tags are required, and artefactual aggregation may trigger false positive signals. Similarly, Y2H systems can screen stable, strong, and direct protein–protein interactions (Brückner et al., 2009). Isothermal titration calorimetry has acceptable sensitivity for interaction and thermodynamic studies and kinetic measurements (Rajarathnam and Rösgen, 2014); however, large quantities of proteins are required. In addition, transient interactions involving low-affinity proteins or an interaction complex with more than two protein components cannot be detected. Furthermore, these methods cannot be used for conducting time-course studies on plant hormones and elucidating the dynamic interactions of signaling components.

Single-molecule fluorescence methods have provided many new insights into the biological processes dominated by macromolecules. In contrast to ensemble-averaged measurements, single-molecule measurements not only describe the real-time conformational dynamics of individual molecules but also identify details of protein–protein or protein–environment interactions that are undetected in ensemble-averaged experiments. For example, studies on the G protein-coupled receptors of the mammalian signal transduction pathway have benefited from various single-molecule methods, facilitating the demonstration of the oligomerization state and mobility dynamics of G protein-coupled receptors within the cell membrane and observation of the conformational transitions of the distinct ligand binding–related states. This crucial information is buried in ensemble-averaged measurement and cannot be effectively extracted from traditional experimental data (Tian et al., 2017). Similar to mammalian G protein-coupled receptors, plant hormone–related receptors in signaling pathways have also attracted increased attention; however, single-molecule experiments were found to be relatively challenging because of the thick plant cell walls and the lack of appropriate imaging techniques (Wan et al., 2011). Currently, single molecule–based quantification methods for plant hormone–related receptors are emerging rapidly (Wang et al., 2015; Bücherl et al., 2017).

Previous studies have mainly focused on plasma membrane receptors, and several details of the complete signaling pathway inside living cells, particularly regarding the downstream signal transduction components, remain unknown (Clouse, 2011). Alternatively, *in vitro* single-molecule methods are feasible for identifying the key points in signaling pathways because *in vitro* experiments suppress the long-distance transportation of signal transduction components between different organelles in living cells and restrict the target motion range near the slide surface. The most common *in vitro* single-molecule assays are usually performed using conventional total internal reflection fluorescence microscopy (TIRFM).

Various single-molecule methods have been developed for the detection of different fluorescent observables. The oligomerization state of functional molecules and stoichiometry of different components within a complex can be quantified using the stepwise photobleaching of fluorescence trajectories (Ulbrich and Isacoff, 2007; Jiang et al., 2011). When this technique is combined with super-resolution microscopy, a single fluorophore can be localized with a precision of down to 10 nm, which can determine the spatial alignment of different subcomponents within one dense structure and the true size of the fine structures blurred by the diffraction limit (Hell and Wichmann, 1994; Betzig et al., 2006; Rust et al., 2006).

To ascertain intramolecular conformational changes or intermolecular interactions, single-molecule Förster resonance energy transfer (smFRET) (Förster, 1948; Clegg, 1992) may be a suitable method. Energy transfer efficiency represents the distance between FRET donor and acceptor, resulting in a smFRET resolution of 3–10 nm, which is even less than the spatial resolution achieved using super-resolution microcopy (Ha et al., 1996). Alternating laser excitation (ALEX) is an auxiliary technique used to distinguish whether low smFRET efficiency is caused by a large distance or a missing acceptor. In principle, ALEX uses two excitations to alternately excite the donor and acceptor. Three observables, namely the respective emissions of two fluorophores and the smFRET signal, yield the stoichiometry ratio and FRET efficiency simultaneously (Kapanidis et al., 2004). In plant hormone signal transduction pathways, the membrane or transmembrane receptors exhibit specificities and diversities. The receptors are the portals of these pathways and play a crucial role in distinguishing different hormones and transducing signals into the cell. However, the exact mechanisms through which receptors recognize their ligands and subsequently switch on or off their functions are under investigation. Furthermore, the mechanism mediating the conformational changes in receptors after associating with plant hormones has remained unknown. By detecting nanoscale conformational changes as well as ligand binding events, the smFRET dynamics of macromolecular machines can be revealed (Juette et al., 2014).

Macromolecules in the natural state are always in motion. Some studies have suggested that the experimental outcomes of immobilization procedures or single-molecule trapping, such as anti-Brownian electrokinetic (ABEL) trap (Cohen and Moerner, 2006; Fields and Cohen, 2011), may not represent the true behaviors of individual molecules. To avoid any unfavorable effects, the characteristics of freely diffusing molecules are being measured. Real-time single-particle tracking with wide-field fluorescence microscopy (Saxton and Jacobson, 1997) is the most commonly used method for characterizing diffusion. Diffusion parameters can be obtained by analyzing tracked molecules within an adjacent image frame series. Fluorescence correlation spectroscopy (FCS) with confocal microscopy has also been frequently used for diffusion measurements (Haustein and Schwille, 2007). It allows the correlation analysis of fluorescence intensity fluctuations, which may be derived from many fluorophore factors within the detection volume, including diffusion and concentration. Therefore, experimental data analysis can provide the diffusion coefficients of individual tagged molecules.

In this paper, we describe novel single-molecule fluorescence methods for exploring plant hormone signal transduction pathways. The paper explains the experimental design and data analysis and validates the method in several manners: using the stoichiometry of a light-dependent plant signaling protein, through a test of the fluorescent protein's fluorescence state under different oxygen environments, and identifying the competitive association with substrates among proteins in the BR signaling system.

## Materials and methods

### Chemicals and reagents

For plasmid construction, universal or specific primers were obtained from Sunny Biotechnology Company (China). DNA polymerase KOD Plus-Neo was purchased from TOYOBO (Japan). DNA restriction enzyme, T4 ligase, and DNA marker were purchased from Takara (China). Top10 competent cells were purchased from TianGen (China). Agar powder (>99.5%, AR) and 50 × TAE buffer (>99.5%, AR) were purchased from Sangon (China). Plasmid miniprep, DNA gel extraction, and polymerase chain reaction clean kits were purchased from Axygen (China). To enable protein expression and purification, tryptone (>99.5%, AR) and yeast extract (>99.5%, AR) were purchased from BBI (China). *Escherichia coli* BL21 (DE3)-PLysS and *E. coli* BL21-CodonPlus (DE3)-RIPL competent cells were purchased from 2nd lab (China). Antibiotics, such as ampicillin, were purchased from Solarbio (China). Affinity and Mono-Q columns were purchased from GE health (Sweden). Organic dyes, such as Alexa Fluor 555 C2 Maleimide, were purchased from Invitrogen (A20346, America). SulfoLink coupling resin was purchased from Thermo Fisher Scientific (20401, America). For slide cleaning and passivation, Na_2_S_2_O_4_, concentrated sulfuric acid, 30% H_2_O_2_, and acetone with guaranteed grades (>99.9%) were purchased from SCRC (China). 3-Aminopropyl-triethoxysilane (APES) was purchased from Aladdin (A107147, China), and Methoxy poly(ethylene glycol) succinimidyl carboxymethyl ester (mPEG-SCM) and biotin p-(ethylene glycol) succinimidyl carboxymethyl ester (biotin-PEG-SCM) were purchased from Biomarik (5675-5K and 5472-4K, respectively, China). Streptavidin was purchased from Amresco (E497, America), and the anti-His-tag antibody was obtained from Rockland (600-406-382, America). For flow cell construction, cover slips (25 mm × 25 mm, Fisher) and a 0.3-mm dual adhesive tape (3M, 467) were used. Poly(dimethylsiloxane) (PDMS) was obtained from Dow Corning (Sylgard-184, America). BODIPY-FL-ATP was purchased from Thermo Fisher Scientific (A12410, America).

### Microscopy

The single-molecule methods are based on TIRFM. We used a homebuilt TIRFM that was based on a previously reported design (Friedman and Gelles, 2012) for multicolor imaging. Two excitation lasers (wavelengths 488 and 532 nm) shaped by a beam-expander, dichroic, doublet lens are focused on the back focal plane of the objective (Olympus, APON 60XOTIRF, Japan) using a tiny mirror (Edmund, 2-mm Diameter 45° Rod Lens Aluminum-Coated, 54-092, America). The focused beam passes through the objective and illuminates the sample immobilized on the slide. The reflected laser beam is directed out of the beam path using another tiny mirror placed symmetrically. The signals are collected by the same objective without the obstruction of the tiny mirror pair. The images are separated into two channels using DV2 (Photometrics, DV2) and finally projected onto an electron multiplying charge-coupled device (EMCCD, Andor, DU897E-13CS0-#BV, England). A homemade airtight flow cell was mounted on the TIRFM for air-controlled experiments.

### Expression plasmid construction

For *in vitro* TIRF experiments, at least one of the proteins of interest must be immobilized on the cover slip. The most common technique for achieving this is immobilization through streptavidin–biotin linkage (van Oijen et al., 2003) For monomeric proteins, the linkage was completed using anti-His-tag antibodies, and vectors such as pET-28a were used to express, purify, and immobilize through His-tag. For other proteins investigated in the same experiment, pMAL-C2X or pET-42a vector was used to express and purify through MBP-tag or GST-tag. For fusion proteins, 6–10 amino acid linkers were inserted between their sequences. To assess protein dimerization and stoichiometry, antibodies should not be used for linkage; instead, vectors with StrepII-tag or avidity vectors, such as pET-52b or pAN/C vector, must be used. One-to-one stoichiometry linkage was achieved using bivalent streptavidin, the preparation of which is described in subsequent sections.

### Protein expression and purification

**Figure 1:**
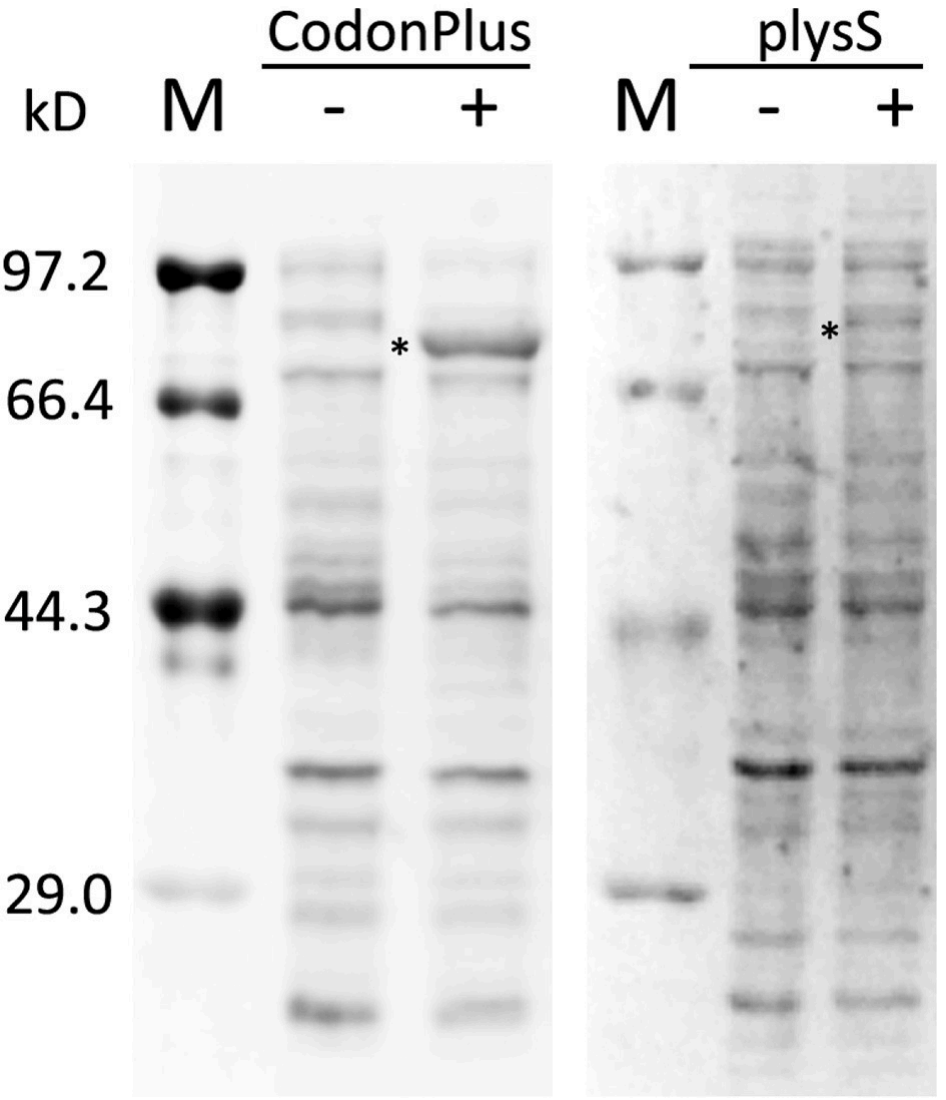
Expression check of His-MBP-BES1 expressed in BL21-CodonPlus(DE3)-RIPL and BL21(DE3)plysS. Same plasmid, containing sequence of His-MBP-BES1, was transformed into competent cells and expressed in 37°C for 3 h with (+) and without (–) 1 mM IPTG induction. The expression levels were analyzed by protein electrophoresis in two gels. The expression level in BL21-CodonPlus(DE3)-RIPL was improved significantly compared with BL21(DE3)plysS in the same condition. M, protein marker; –, without IPTG; +, with 1mM IPTG. ^*^, His-MBP-BES1 band.

Vectors containing His-MBP-BES1, MBP-BIN2, His-BIN2-eGFP, MBP-BKI1C, and GST-JKC were transformed into *E. coli* BL21-CodonPlus (DE3)-RIPL competent cells for expression. Differences in codon usage preference among organisms lead to various problems related to heterologous gene expression. Therefore, rare codon analysis must be performed prior to vector selection. Target proteins, whose sequences contain numerous rare codons, are better expressed in *E. coli* BL21-CodonPlus (DE3)-RIPL cells (Figure 1). A culture of transformed *E. coli* BL21-CodonPlus (DE3)-RIPL cells was grown at 37°C in Luria Bertani (LB) medium until an optical density of 0.4–0.6 was achieved at 600 nm. The cell culture was then induced with 400 μM isopropyl β-D-1-thiogalactopyranoside (IPTG), and grown for an additional 10–12 h at 20°C. After expression, purification had to be performed as soon as possible to maintain sample freshness. For purification, lysates were dissolved in a buffer containing 20 mM Tris-HCl (pH 8.0) and 20 mM NaCl and were purified in an affinity chromatography column (amylose, GST, and Ni columns for MBP-tag, GST-tag, and His-tag, respectively) and gel filtration column.

Vectors containing MBP-14-3-3κ/A206K were transformed into *E. coli* BL21 (DE3)-plysS competent cells for expression. The culture of transformed *E. coli* BL21 (DE3)-plysS cells was grown at 37°C in LB medium until an optical density of 0.4–0.6 was achieved at 600 nm. Subsequently, the cell culture was induced with 400 μM IPTG, and grown for an additional 6 h at 37°C. For purification, lysates were dissolved in a buffer containing 20 mM Tris-HCl (pH 8.0) and 20 mM NaCl and were purified in amylose and gel filtration columns.

Homemade vectors with *aCry-mOrange2* were transformed into *E. coli* BL21 (DE3) cells. Recombinant aCry-mOrange2 proteins were expressed as StrepII-fusion and His-fusion proteins in *E. coli* at 20°C. For the purification of the recombinant proteins, lysates were purified through Ni^2+^ affinity, anion-exchange, and size-exclusion chromatography. The flavin adenine dinucleotide redox states in aCry-mOrange2 proteins were monitored and maintained in oxidized states after each purification step.

Expression and purification method of His-AK can be found in the literature (Tan et al., 2009).

After purification, the proteins labeled with fluorescent proteins were subjected to cover slip immobilization. For proteins designed to be labeled by organic dyes, the following additional steps (Tan et al., 2014) were performed. The concentrated target proteins (50–100 μM/100 μL) were mixed with 1 μL of dye (1 mg of dye powder dissolved in 100 μL of dimethylsulfoxide) in the dark at room temperature for approximately 3 h. The labeled and unlabeled proteins were separated from free dye molecules in a gel filtration column in the dark. Because of minor differences in the molecular weights of labeled and unlabeled proteins, gel filtration was unable to separate these proteins. Therefore, SulfoLink coupling resin was used, which absorbs unlabeled proteins by forming disulfide bonds with free Cysteine (Cys) residues in a protein mixture. The sample was mixed with 100 μL of resin in a slow rotating mixer at 40 rpm in the dark at room temperature. The sample was washed using a protein buffer, and the resin and sample were separated through centrifugation at 13,000 rpm. The washing and centrifugation steps were repeated three times, and the protein supernatant was then collected. After this step, only a low percentage of unlabeled proteins was present. After purification, microscopic observations had to be conducted as soon as possible to maintain the proteins in the freshest state. Usually, microscopic observation must be completed within 3 days or even 24 h, which is before the degradation of unstable proteins after purification. The methods used to probe the functional integrity or degradation of purified proteins were specific to each protein under investigation. For example, the functional integrity of a phosphatase-like BIN2 was probed using the level of BES1 phosphorylation.

### Cover slip passivation

Cover slip cleaning and surface passivation were performed as follows (Tan et al., 2014). Cover slips were incubated in a mixture of concentrated H_2_SO_4_ and 30% v/v H_2_O_2_ at the ratio 3:1 for 30 min, followed by thorough rinsing using ultrapure water. Subsequently, the cleaned cover slips were incubated in acetone containing 3% 3-aminopropyltriethoxysilane for 30 min, followed by thorough rinsing using ultrapure water and drying using clean nitrogen gas. The PEG solution was prepared by dissolving 4.5 mg of mPEG-SCM and 0.5 mg of biotin-PEG-SCM powder in 200 μL of 0.1 M NaHCO_3_. Finally, 50 μL of the PEG/biotin-PEG solution was incubated between two cover slips for 3 h at room temperature. After cover slip passivation, the slide surfaces were coated with a PEG layer with randomly distributed functional biotin terminals.

### Flow cell construction

**Figure 2:**
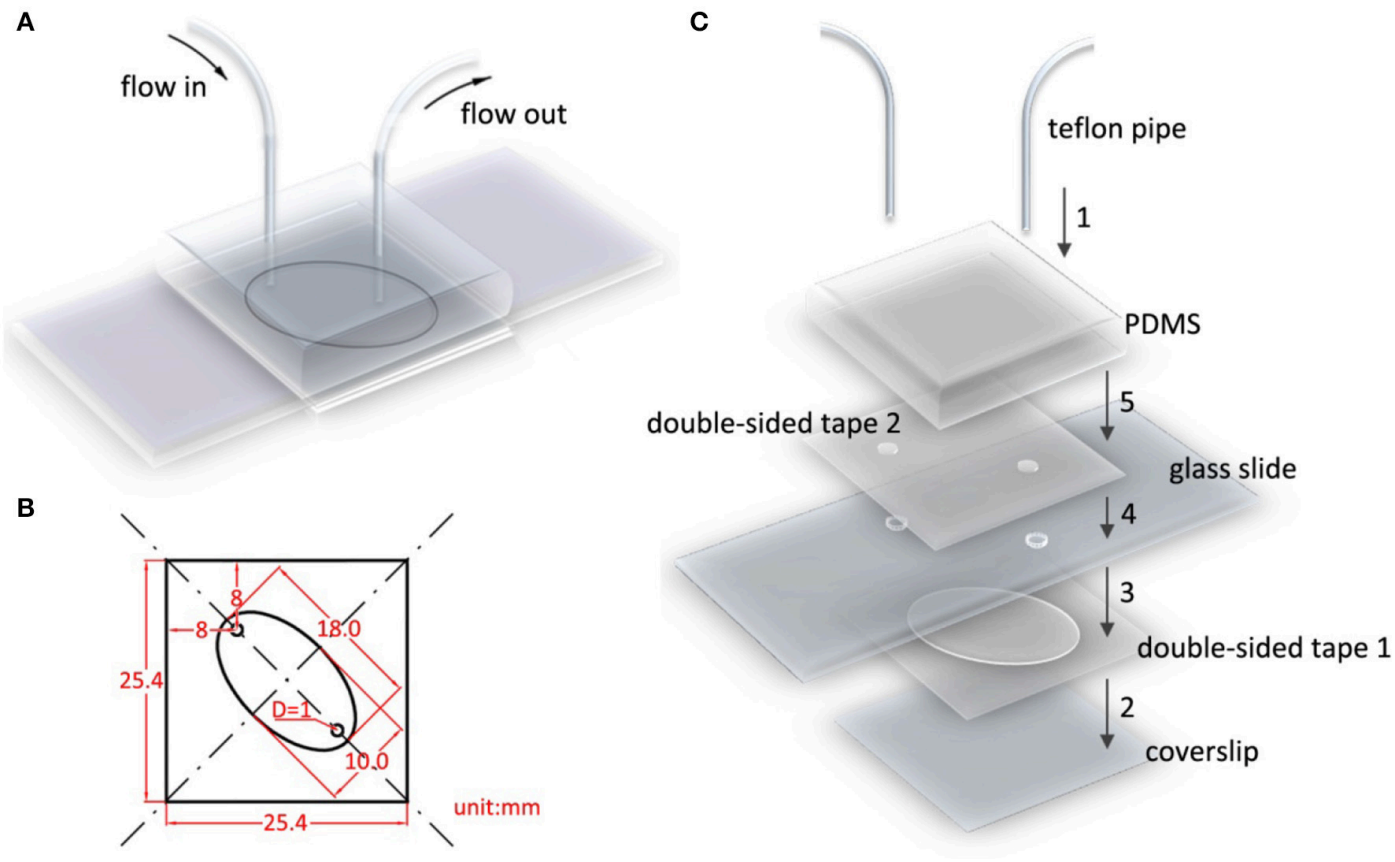
Flow cell model and assembly diagram. **(A)** Flow cell model. **(B)** Detailed geometry of double-sided tape 1, 2, with respect to the holes drilled on glass slide. The elliptical hole is the flow cell chamber. **(C)** Flow cell is assembled in five steps. Step 1, mount Teflon pipes on PDMS. Step 2–5, successively and securely adhere double-sided tape 1 with coverslip, glass slide, double-sided tape 2, PDMS. The position of two pipes, holes in double-sided tape 2, and glass slides should be precisely aligned.

Figure 2A presents an assembled flow cell model. Double-sided tape, glass slides, and PDMS were prepared before assembling the flow cell. Double-sided tape 1 (Figure 2C) was designed to adhere the cover slip and glass slide and simultaneously leave an elliptical hole as the flow cell chamber. The detailed sizes are displayed in Figure 2B; the two 1-mm diameter circles are holes that were punched and drilled on double-sided tape 2 and the glass slide, respectively. In addition, a piece of 25 by 25 by 10 mm^3^ PDMS with a flat smooth bottom surface was prepared to mount Teflon pipes. The glass slide and cover slip were cleaned and passivated in advance. Because the glass slide was not designed to immobilize proteins, a PEG layer was coated on its surface. The flow cell was assembled in five steps. First, the two pipes were mounted using PDMS (Figure 2C, step 1). The position of the two pipes exactly matched the two holes on the glass slide. The pipes mounted with PDMS are reusable. Subsequently, the cover slip and two-hole glass slide were tightly attached using double-sided tape 1 (Figure 2C, steps 2 and 3). Finally, the glass slide and PDMS were tightly held using double-sided tape 2 (Figure 2C, steps 4 and 5).

### Protein immobilization and stoichiometry regulation

**Figure 3:**
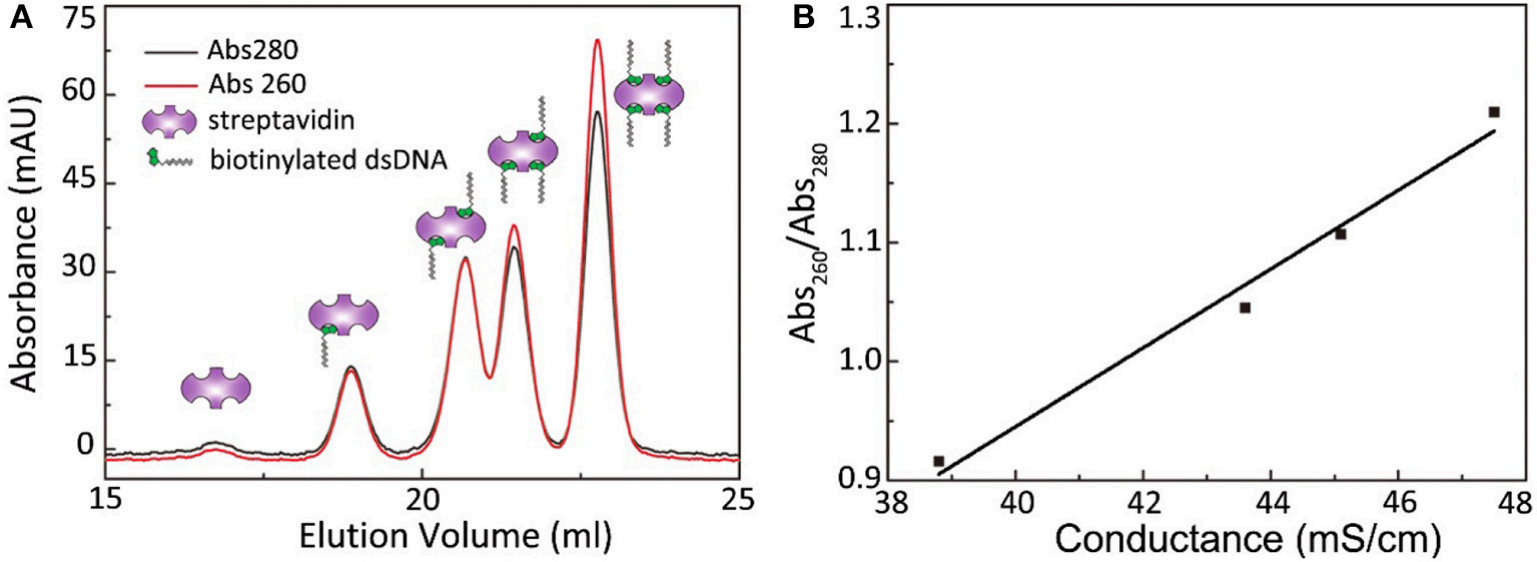
Separation of streptavidin with different valence by Anion exchange chromatography. **(A)** The absorbance curves at 260 and 280 nm of streptavidin with different valence. **(B)** The 260 nm/280 nm absorbance ratio scales linearly with the peak conductance.

A passivated cover slip embellished with PEG and biotin-PEG was incubated with 0.5 μL of streptavidin (stock solution concentration, 10 mg/mL) dissolved in 0.5 mL of buffer for 15 min. In addition, 0.5 mL of His-tag fusion protein (concentration, approximately 50 pM−1 nM) was incubated with 1 μL of biotinylated His antibody (stock solution concentration, 1 μM) in a sample tube for 15 min. After thoroughly rinsing the cover slip with a buffer solution, the protein/antibody solution was transferred onto the cover slip and incubated for another 15 min, followed by thorough rinsing using 1 mL of protein buffer. When using a flow cell, the aforementioned procedure can be performed using a syringe pump and piping. Moreover, a simple CoverWell profusion chamber can be used with a drop (approximately 30 μL) of protein buffer on the cover slip (Tan et al., 2014). A sample is ready for microscopic observation after these procedures. If protein dimerization or stoichiometry is under investigation, the proteins can be fused with StrepII-tag or Avi-tag for direct linkage without the biotinylated His-tag antibody. Furthermore, bivalent streptavidin (Sun et al., 2014) were prepared to eliminate the possibility of additional binding sites on regular streptavidins by using the following procedure: the mixtures of streptavidin and biotinylated DNA were incubated for 0.5 h at room temperature at a molar ratio of 1:2. Streptavidins with different valences were separated through Mono-Q anion-exchange chromatography under alkaline conditions (optimal pH 8–9; Figure 3).

## Results and discussion

### Experimental design

#### General considerations

**Figure 4:**
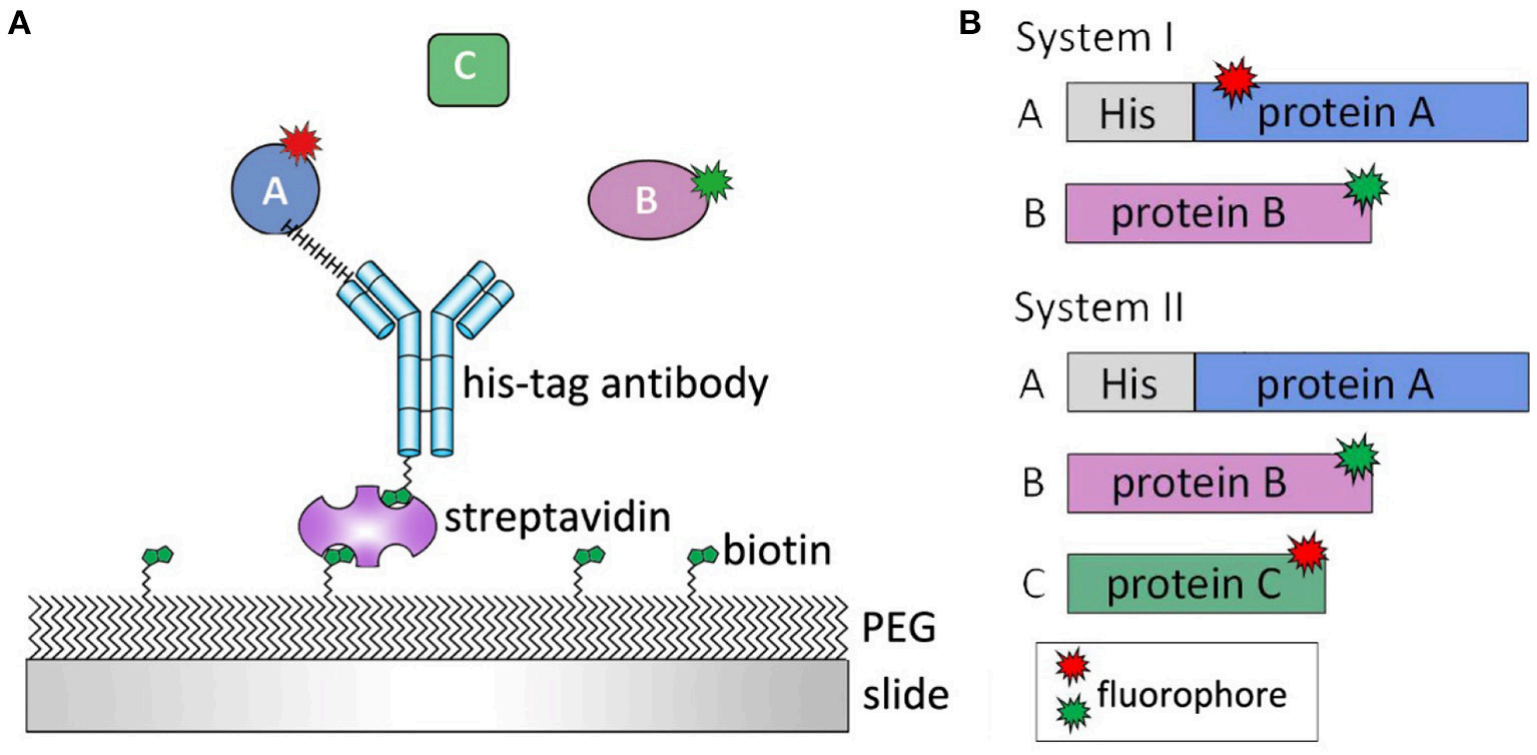
Single-molecule experimental design. **(A)** Clean slides are passivated by poly-ethylene glycol (PEG) and biotin-PEG to eliminate unspecific protein bindings. Protein A is immobilized on slide surface by biotin-streptavidin-antibody linker. Protein B or C is then added and their interaction is monitored in real-time. **(B)** For a two-protein system (System I), both proteins are labeled by fluorophores, and the protein with His tag is immobilized on slide surface. For a three-protein system (System II), protein A, which connects protein B and C, is immobilized on slide surface with His tag. Both protein B and C are labeled so the interactions between three proteins can be observed.

In the employed TIRFM-based single-molecule fluorescence technique, if the target proteins are moving freely in the buffer, the dwell time of the target protein flowing through the focal plane is shorter than common camera frame rates. Therefore, little information can be effectively captured. To maintain focus on the target proteins and their interaction events, the specimens had to be immobilized on a glass slide (Figure 4A; van Oijen et al., 2003). For systems involving more than one protein, such as a phosphatase associating with its substrate or three proteins interacting in a certain sequence to relay signals, a general protein construct design is required, as shown in Figure 4B. Usually, one protein (protein A) is immobilized on a slide and another protein (protein B) is added, followed by incubation or immediate observation. On the basis of the expected interaction cascade, a protein related to all other proteins is most suitable for immobilization. Moreover, a stable signal from protein B can only be observed when the interaction occurs between protein B and surface-tethered protein A. To study different systems, experiments can be operated in different manners. For example, to investigate the interaction strength of two proteins regulated by a certain ion, protein B can be added on the slide embellished with protein A under the condition of ion titration to observe the binding percentage or dynamics of proteins A and B. The interaction readout can be in the format of colocalization single-molecule spectroscopy (CoSMoS) (Friedman and Gelles, 2012), FRET (Förster, 1948; Clegg, 1992), or BiFC (Bimolecular fluorescence complementation) (Hu et al., 2002).

#### Labeling strategy

Most proteins are nonfluorescent; therefore, they must be labeled by fluorophores for single-molecule fluorescent interaction or dynamic assays. The selection of appropriate fluorophores as labels is a critical step in target protein construct design. Fluorescent proteins and organic dyes are the most popular labeling methods in single-molecule fluorescence microscopy. For proteins with few Cys residues or a known structure, organic dyes are preferred because of their small size and flexibility. The labeling sites should be carefully selected when using organic dyes. The most common linkage is the conjugation of the maleimide derivative of organic dyes with the thiol group of proteins (Allewell et al., 2013). Therefore, the number and sites of Cys residues are critical factors. To assess protein–protein interaction stoichiometry, one protein molecule should only be labeled by one dye molecule. Mutations should be introduced to eliminate additional Cys residues such that only one Cys residue in each molecule is available for labeling; this residue must be accessible from the buffer solution to the target protein structure. The mutations of Cys residues are typically selected on the basis of homologous sequence alignment and structure analysis.

When the structure of the protein of interest is unknown or the protein sequence has many Cys residues, fluorescent proteins must be used as probes to avoid generating many mutations that disrupt the native protein function. The sequence of fluorescent proteins can be fused to the N terminal or after the C terminal of the target proteins. This labeling method has two advantages: one-to-one labeling efficiency and compatibility with *in vivo* usage. With regard to their size, fluorescent proteins may affect the function of the corresponding terminal or block sites of interaction with other proteins. Therefore, a linker between the target and fluorescent proteins is necessary. In addition, fluorescent proteins must not be linked to the functional terminal. Control experiments are required to assess whether the fusion proteins retain their original function. If neither of these labeling methods are successful, unnatural amino acids with amber codons or a chemoenzymatic labeling strategy (Juillerat et al., 2003; Gautier et al., 2008; Tian et al., 2017) is an alternative method.

#### Determination of fluorophore colors

The colors of fluorophores are mainly determined by the microscope used for the experiment. For a dual-color-labeled system, we require lasers of two colors for excitation and two signal channels for detection. The fluorophore excitation and emission spectra must cover the laser wavelength and signal channel detection range, respectively. In addition, the emission spectra of two fluorophores must not substantially overlap because this would contribute to the background noise and reduce the signal-to-noise ratio (SNR). For example, for a microscope equipped with 488- and 532-nm excitation lasers, the fluorophore pair of GFP/mOrange2 or alexa488/alexa555 can be used.

### Method validation

#### Protein sample preparation for detection

TIRFM-based single-molecule experiments are extremely susceptible to environmental contamination. Because of the use of ultrasensitive detectors, even tiny nonfluorescent dust particles on the slide surface can generate troublesome background noise through light scattering. Therefore, cover slips should be thoroughly cleaned before microscopic observation. In addition, impurities can be generated during the surface passivation step. Therefore, before each set of data is acquired, a clean slide without protein samples should be tested. The total number of impurity spots in the camera frame must be restricted to within 5% of the molecule number observed in a regular experiment.

To achieve protein immobilization, purification, and fluorophore labeling, tags or mutations are introduced to modify the target proteins. These operations may cause structural change or hinder functional activity; therefore, control experiments must be conducted to assess target protein activity.

#### Single-molecule monomer regulation and dimer detection

Limited by diffraction, the resolution of the TIRF microscope is approximately 200 nm. This is almost 10 times regular protein sizes. Therefore, for distinguishing individual proteins using TIRFM, their spacing should be at least larger than 200 nm; otherwise, massive overlapping spots will be observed. Consequently, for single-molecule experiments, labeled proteins are usually diluted to pM–nM concentrations before microscopic observation. However, achieving such low concentrations across different batches of protein samples is difficult. In addition, each streptavidin has four biotin-binding sites; therefore, more than one target protein may be linked to one streptavidin, and multiple and overlapping bright spots may obscure data interpretation. This complication is particularly evident in smFRET experiments because their readouts are based on relative intensities. To resolve this problem, modified streptavidin with only two binding sites can be used. This modification is performed using anion-exchange columns that prepare multivalent streptavidin (Sun et al., 2014). Streptavidin with two dsDNA can be used for linkage because of the prior blocking of the two biotin-binding sites.

**Figure 5:**
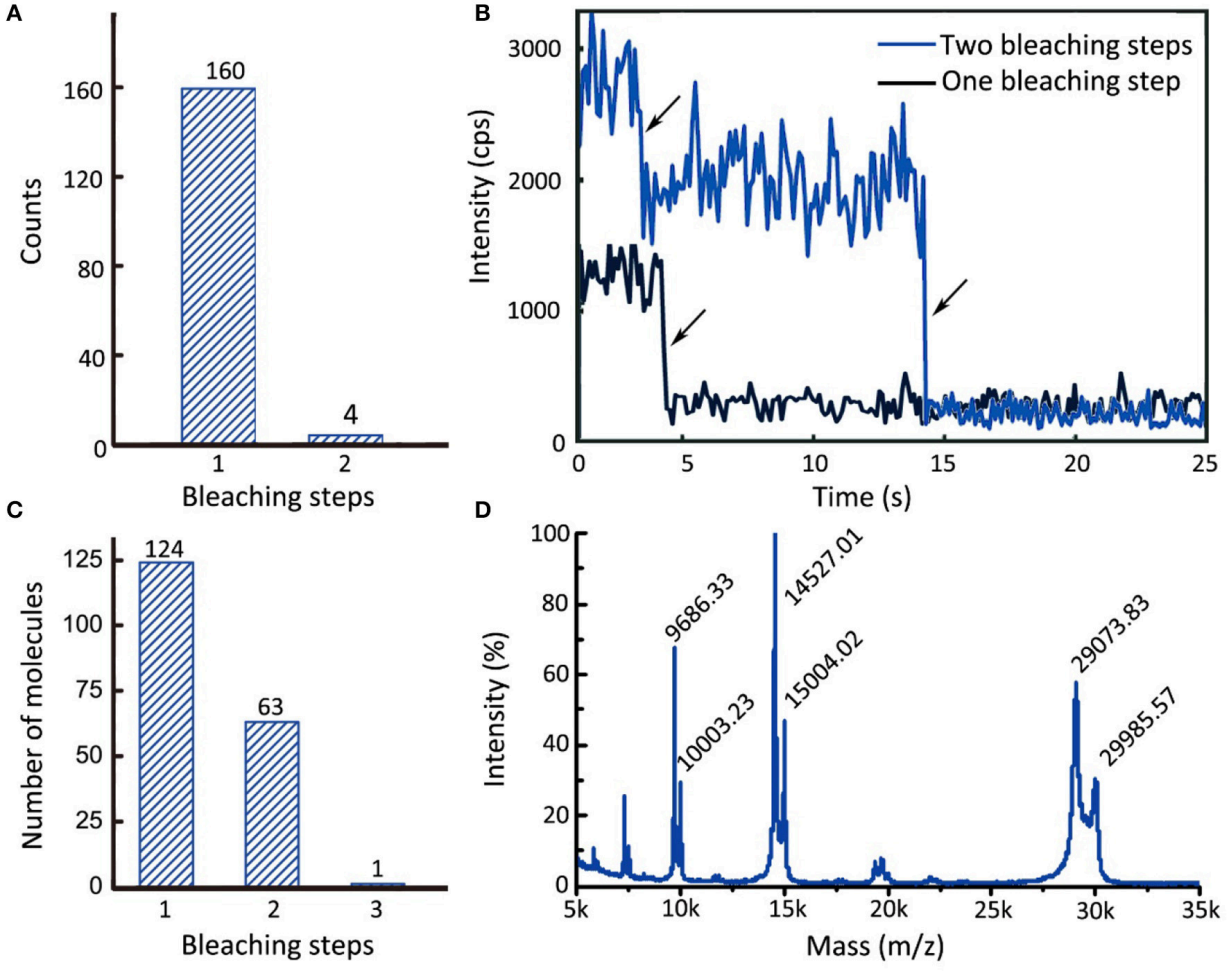
Single-molecule monomer regulation of Strep-aCry-mOrange2 and the detection of 14-3-3κ stoichiometry. **(A)** The accumulated molecule counts of single and double bleaching steps of Strep-aCry-mOrange2. **(B)** Trajectories of Strep-aCry-mOrange2 with different bleaching steps. **(C)** The accumulated molecule counts of different of bleaching steps of 14-3-3κ-alexa555. **(D)** Mass-spectrum of 14-3-3κ-alexa555. Molecular weight of 14-3-3κ: 29073.70D. Molecular weight of maleimide derivative of alexa555: ~ 1,250 (The structure of alexa555 hasn't been announced yet). The relative intensity of peak 29073.83, which represents unlabeled 14-3-3κ, is 56.9%. The relative intensity of peak 29985.57, which represents labeled 14-3-3κ, is 30.3%. The labeling efficiency *E* = 30.3%/(56.9% + 30.3%) = 34.7%.

The animal-like cryptochrome (aCry) of *Chlamydomonas reinhardtii* is a dimerization-prone plant signaling protein (Oldemeyer et al., 2016). In the present study, we used a single-molecule assay to demonstrate that, with careful sample handling, aCry molecules can be maintained in their monomeric state by using divalent streptavidin immobilization. In this experiment, aCrys with an N-terminal StrepII-tag fused with C-terminal monomeric mOrange2 was immobilized on a slide through divalent streptavidin and then monitored under a TIRF microscope. Analysis of the fluorescence trajectories of aCry-mOrange2 revealed that nearly all aCry-mOrange2 proteins underwent one bleaching step (Figure 5A). Less than 2% of the overall trajectories exhibited two bleaching steps (Figure 5B, blue trajectory), indicating the presence of a dimer or more than two aCry-mOrange2 molecules within the bright spot. These results indicated that the aforementioned procedure can be used to prepare monomeric aCry molecules.

The 14-3-3 proteins form a large protein family that has been extensively studied in both plant and other eukaryotic systems (Aitken et al., 1992). They can associate with phosphorylated client proteins to modulate their function (Denison et al., 2011). In sodium dodecyl sulfate–polyacrylamide gel electrophoresis, 14-3-3 proteins appear in a dimerized protein band (Aitken et al., 1992). In the present study, single-molecule methods were used to observe the active form of 14-3-3κ when bound to phosphorylated BES1 (pBES1). In this experiment, BES1 with a His-tag, which was incubated with BIN2 for 30 min at 37°C in a phosphorylation buffer (Ryu, 2013), was immobilized on a slide. Subsequently, alexa555-labeled 14-3-3κ/C103S was added, and the interaction between 14-3-3κ/C103S-alexa555 and pBES1 was observed under a TIRF microscope. In the 188 observed trajectories, one, two, or three bleaching steps were recorded (Figure 5C). Because the labeling efficiency of 14-3-3κ with alexa555 was 34.7% (from matrix-assisted laser desorption/ionization–MS; Figure 5D), the likelihood of 14-3-3κ functioning as a dimer was the highest among all probable models. Molecules with three bleaching steps may be due to two or three molecules appearing under the same pixel. This single-molecule experiment revealed the active dimeric form of 14-3-3κ.

#### Flow cell for plant hormone signal transduction pathway investigation

In plant hormone signal transduction pathways, gas-phase molecules such as ethylene or some reactive oxygen species are also very important factors for regulating and controlling signal transduction (Apel and Hirt, 2004; D'Autréaux and Toledano, 2007). Under different gas concentrations, cells may have different responses. However, in the conventional experimental setting, it is difficult to control gaseous conditions *in vivo* or *in vitro*. In our method, we constructed a flow cell to maintain an airtight environment during microscopic observation. All buffers and samples were sealed in the flow cell system to prevent air exchange. Figure 2 presents a diagram of the easy homemade flow cell model. The edge of the cover slip and glass slide substrate were attached using 0.3-mm double-sided tape, leaving an elliptical center for buffer flow and an observation chamber. The holes for liquid handling were drilled on the glass slide substrates. The buffer was fed through Teflon piping, with inlets mechanically supported by PDMS, entered through the elliptical chamber, and exited through the other hole. The amount of gas dissolved in the buffer solution could be controlled at the buffer reservoir and monitored in real time. Using this system, the gas concentration could be fixed or even titrated during observation. In addition, for systems involving multiple protein interactions, proteins could be added consecutively to control reaction sequences, conduct small-scale stop-flow experiments, and observe interactions in real time.

**Figure 6:**
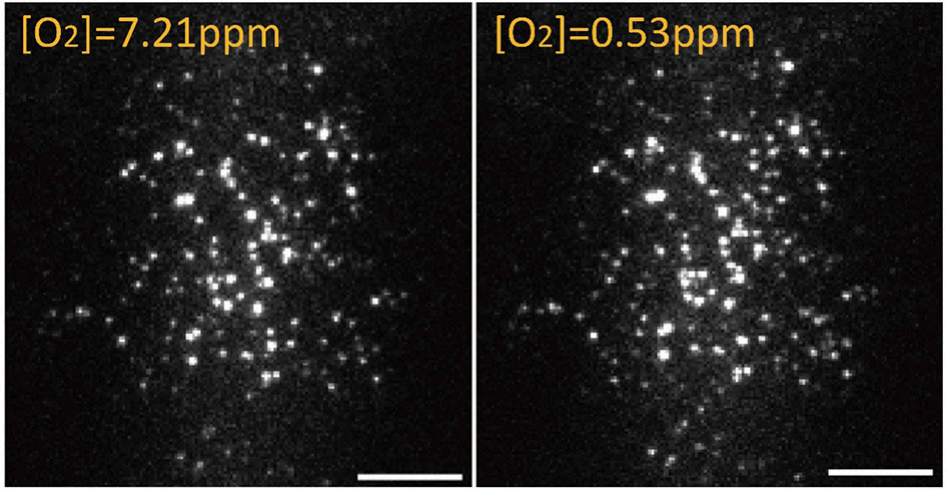
On the same area, 106/116 = 91% eGFP/A206K spots remain fluorescent when oxygen concentration was adjusted from [O_2_] = 7.21 ppm to [O_2_] = 0.53 ppm. Scale bar: 10 μm.

The activation or intensity of some fluorescent proteins is affected by the environmental oxygen (Tsien, 1998). The present study used a single-molecule assay with a flow cell to determine whether eGFP exhibits different responses under variable oxygen concentrations (Vordermark et al., 2001). By using a flow cell, we depleted O_2_ from 7.21 to 0.53 ppm and enumerated the fluorescence spots and intensity of eGFP/A206K in the same view (Figure 6). Because the original eGFP can form a weak dimer, the monomeric form of eGFP/A206K was used as a test sample. The results indicated that 106/116 = 91% of spots remained bright when the oxygen concentration was decreased from 7.21 to 0.53 ppm. This slight decrease may be attributed to photobleaching during the observation period. This experiment demonstrated that a normal environmental oxygen concentration does not affect the fluorescence intensity or florescent state of eGFP/A206K.

#### Study of the competitive association of phosphorylated BKI1 and pBES1 with 14-3-3κ using CoSMoS

CoSMoS (Friedman and Gelles, 2012) is usually used to investigate the binding and dissociation times and kinetics of multiple components or to map the distribution of two or more components in cells. Unlike traditional biological methods, CoSMoS can capture real-time protein interaction dynamics. For example, if two labeled molecules, molecules 1 and 2, are extremely close to each other and associating directly or binding with the same molecule, the spots of these molecules coincide when observed through an EMCCD, indicating a direct or indirect interaction between them. However, limited by diffraction, CoSMoS is unsuitable for observing molecules at high concentrations. Moreover, only when the locations of two or more molecules are superpositioned at an extremely low density, such as <1 molecule/μm, can an interaction be considered to have occurred among the molecules.

**Figure 7:**
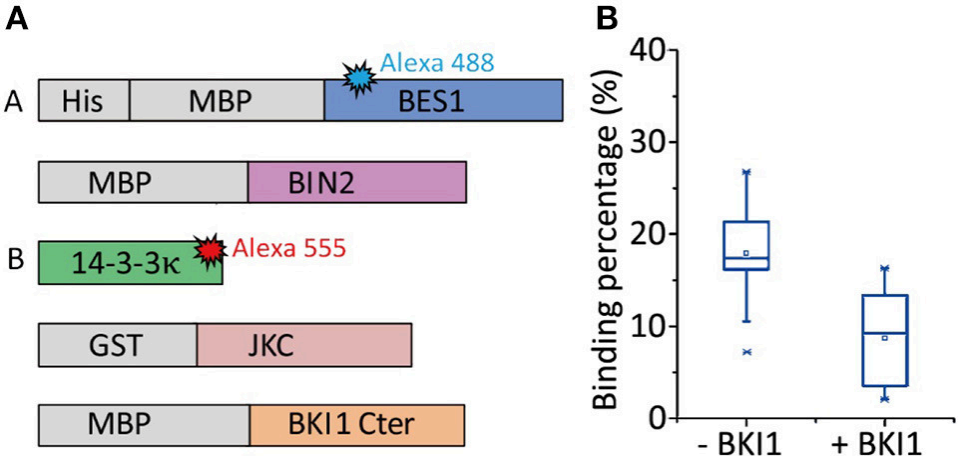
The competitive association of 14-3-3κ- with pBES1 and pBKI1 observed by CoSMoS. **(A)** Protein BES1 with His-tag was immobilized on slide surface. BES1 and BKI1C are phosphorylated by BIN2 and JKC, respectively, in phosphorylation buffer for 1 h at 37°C. The binding percentages of 14-3-3κ with pBES1 were observed before and after incubating with pBKI1C. **(B)** Binding percentage of 14-3-3κ with pBES1 before and after pBKI1C treatment. The percentage only decreased by half, from (18 ± 5)% to (9 ± 5)%, even though we used a very high concentration pBKI1C (>1,000 times larger than that of pBES1) and incubated for 1 h.

The present study used the BR signaling pathway (Wang et al., 2012) as an example. The existence of an alternative and possibly faster BR signaling transduction pathway has always been debated. After BR binds to its receptor BR insensitive1 (BRI1), BRI1 phosphorylates BKI1 at its C terminal, and this phosphorylated BKI1 (pBKI1) can also associate with 14-3-3κ (Wang et al., 2011). However, evidence of this direct competition is rare. If pBKI1 can competitively strip 14-3-3κ off the 14-3-3κ-pBES1 complex, pBES1 may be directly imported into nuclei to perform its function as a transcription factor with the help of protein phosphatase 2A. This signaling pathway could respond quickly to BR binding because it involves fewer components. To verify the existence of this faster signaling pathway or whether 14-3-3κ is competitively replaced by pBKI1 on pBES1, allowing pBES1 to perform its transcription function, we used CoSMoS to measure the binding percentage of 14-3-3κ with pBES1 before and after adding pBKI1. Figure 7A presents the experimental design. BES1 and BKI1C (BKI1 C terminal) were phosphorylated by BIN2 and JKC (the intracellular domain of BRI1 containing a juxtamembrane segment, kinase domain, and cytoplasmic portion), respectively, in a phosphorylation buffer (Ryu, 2013) for 1 h at 37°C. The results showed that during this hour, the binding percentage to 14-3-3κ was altered only when the concentration of BKI1 was 1,000-fold higher than that of pBES1 (Figure 7B). Considering that such a wide concentration gap is impossible under normal physiological conditions, the binding of 14-3-3κ with pBES1 is comparably stable. However, unbound 14-3-3κ can readily interact with both pBKI1 and pBES1. Therefore, pBKI1 cannot compete with the 14-3-3κ-pBES1 complex to initiate a faster signaling pathway.

### Data analysis

#### Extraction of intensity–time trajectories

**Figure 8:**
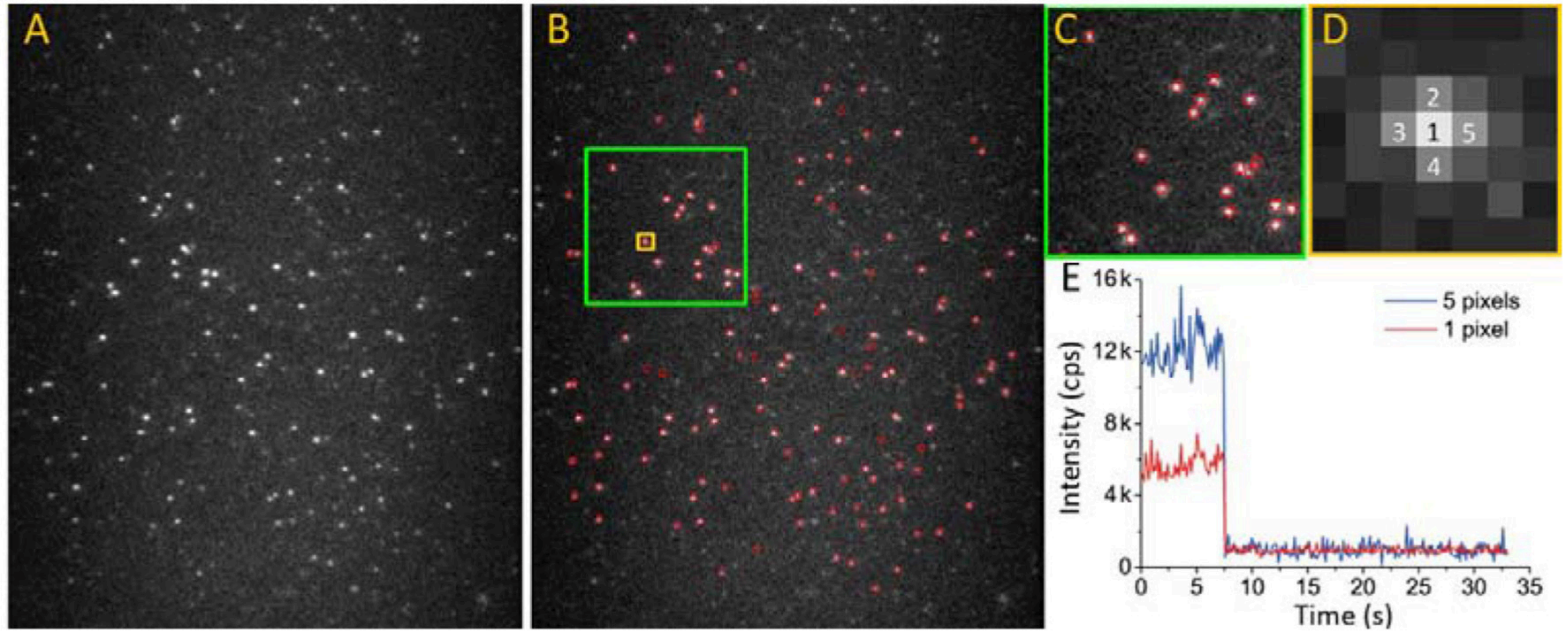
Extracting effective intensity-time trajectory from EMCCD video file. **(A)** 2D image of alexa 555 captured by EMCCD. Excitation laser: 532 nm. Emission spectral range: 500–600 nm. **(B–D)** Applying a signal to background threshold, effective spots (red circles) can be selected. **(C,D)** are enlarged images of the areas marked by green and yellow squares in **(B)**. **(E)** The intensity-time trajectory of a spot analyzed by two different algorithms. Red curve: trajectory of only 1 pixel (pixel 1 in **D**); blue curve: trajectory of intensity summation of five pixels (pixel 1–5 in **D**).

In single-molecule fluorescence methods, the raw data collected by an EMCCD take the form of monochrome video files. The brightness of each pixel corresponds to the observed light intensity. The first step of data processing is screening for effective spots and their intensity–time trajectories in each video. Effective spots are defined as bright spots formed by light emitted from the labeled target proteins instead of background noise or light emitted from impurities on the slide. For regular fluorophores, photobleaching is a typical feature during microscopic observation, which is defined by oxidation-induced irreversible changes in the fluorophore structure. In contrast to photoblinking, photobleaching leads to a permanent nonfluorescent state. A stepwise sharp photobleaching-caused decrease in the intensity in an intensity–time trajectory is the signature of a single molecule (Figure 8E). Before photobleaching and without photoblinking (Moerner, 1997), the fluorescence intensity is typically stable without drastic fluctuations.

Intensity–time trajectory extraction can be completed in four major steps (Figure 8): (1) Estimate the mean background intensity by using at least two images in which all spots are nearly photobleached; (2) Set a threshold above which any bright spot is considered an effective molecule. Use the threshold to estimate the SNR by the background intensity and screen the effective spots. The threshold need not be constant and can be adjusted with time and the microenvironment around the fluorophores; (3) Obtain output intensity–time trajectories. When the SNR of the video is low, the overall data quality can be improved by summing the intensities of the nearest neighboring pixels. Figure 8E shows the trajectories formed by a single pixel or five adjacent pixels; and (4) Apply additional criteria to the effective trajectory pool. For example, identify an abrupt intensity drop indicating single-fluorophore bleaching, rejecting fluorophore blinking or features such as the stability of intensity.

#### Model building and parameter fitting

The single-molecule protein interaction assay used herein is unique because it allows the determination of parameters such as reaction rate constants and stoichiometry. For studying protein–protein or protein–ligand interactions in plant hormone signal transduction pathways, an appropriate model must describe the interaction kinetics. For instance, the reaction of an enzyme associating with or dissociating from its fluorophore-labeled ligand can be described as:

$$\begin{array}{l} E\;+L\;\begin{array}{c} k_{1}\\ ⇌\\ k_{-1} \end{array}\;E\cdot L\\ ↓k_{2}\\ E+L^{b}\;\begin{array}{c} k_{1}\\ ⇌\\ k_{-1} \end{array}\;E\cdot L^{b} \end{array}$$

In this system, fluorophore-labeled enzymes are immobilized on slides and dye-labeled ligands of a certain concentration are added to the buffer to obtain fluorescence information in two channels (two color labels for enzymes and ligands). In the stated equation, *k*_1_ and *k*_−1_ are association and dissociation rate constants, respectively. Considering the photobleaching of labeling fluorophores, a rate constant *k*_2_ was added to this model. *L*^*b*^ is the photobleached ligand. Because enzyme *E* is immobilized on a slide, the observed fluorescence time does not indicate any information of its association or dissociation. In this study, we did not consider the photobleaching of *E*. In principle, the photobleaching of ligands' probe will not interfere with the reaction between the enzyme and its ligand. In this system, *E*·*L* and *E*·*L*^*b*^ are the bound states, and the ligand can only be observed in the bound state before photobleaching.

In this kinetic model, *k*_2_ is a parameter that depends only on the fluorophore photostability and microscopic setup. It can be fitted separately by observing the fluorophore alone. The unbound time, represent the probability of an *L* association event. The bound time was affected by bleaching rate constant *k*_2_ and the dissociation rate constant *k*_−1_. By combining the ligand concentration *L*, fitted *k*_2_, and observed unbound and bound time distributions, the rate constants *k*_1_ and *k*_−1_ can be obtained.

To illustrate how to acquire the rate constants *k*_1_, *k*_−1_, and *k*_2_, we use the enzyme Adenylate Kinase (AK) as an example. Adenylate Kinase (AK) is a well-studied kinase which can catalyze the reaction Mg^2+^· ATP + AMP ⇌ Mg^2+^· ADP + ADP. The aforementioned method was applied to measure the rate constants of AK and ATP association. Alexa555 labeled AK was immobilized on the slide surface via His-tag, 10 nM ATP labeled with BODIPY FL was added through AK buffer, and the interactions are observed on TIRFM. In this case, we can revise the kinetic model into:

$$\begin{array}{l} AK+ATP\;\begin{array}{c} k_{1}\\ ⇌\\ k_{-1} \end{array}\;AK\cdot ATP\\ ↓k_{2}\\ AK+ATP^{b}\;\begin{array}{c} k_{1}\\ ⇌\\ k_{-1} \end{array}\;AK\cdot ATP^{b} \end{array}$$

**Figure 9:**
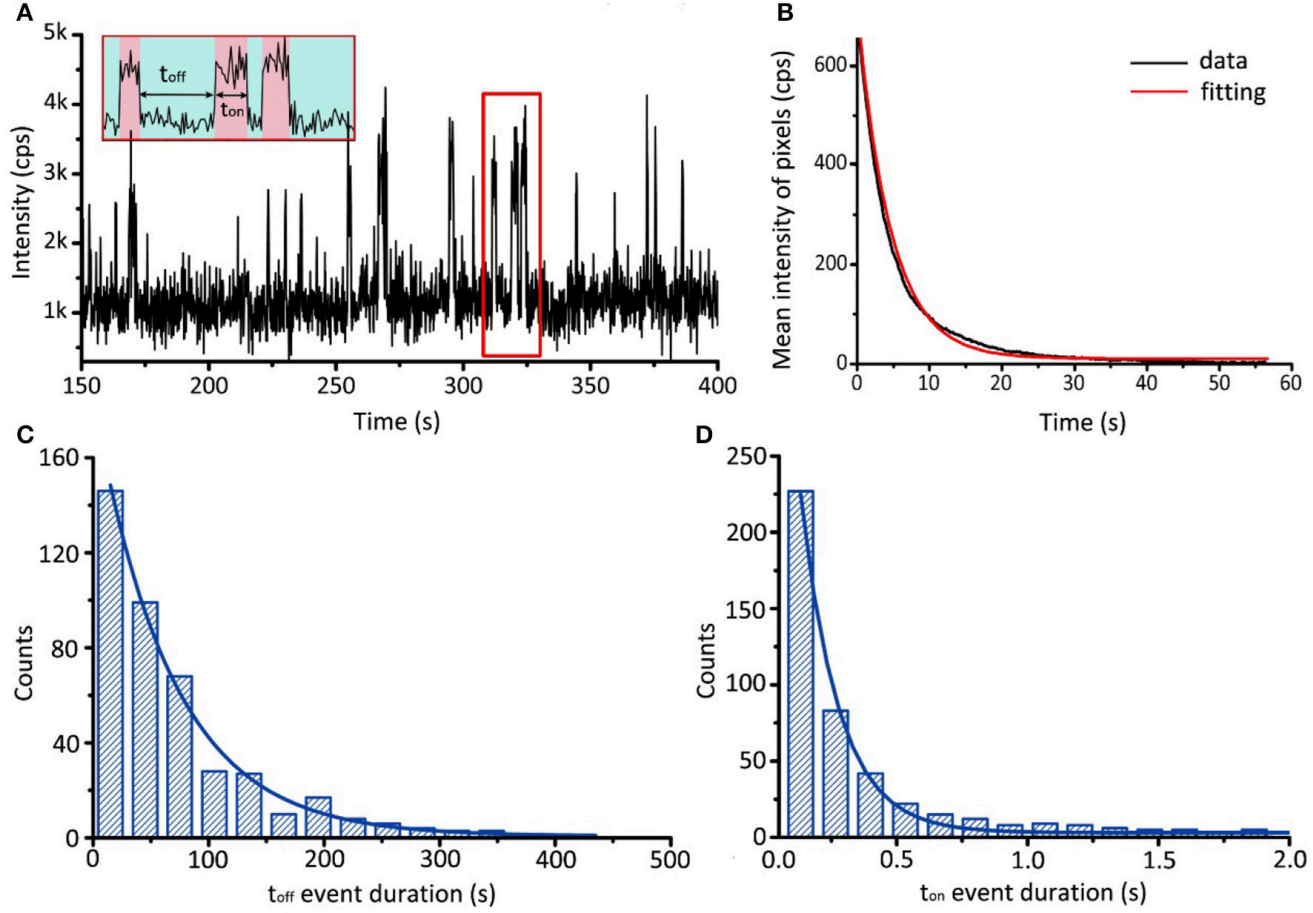
Single-molecule binding assay of AK and ATP. **(A)** Typical fluorescence trajectory of BODIPY FL-ATP associating and dissociating with AK. Association and dissociation are indicated by red and green bands, respectively. **(B)** Evolution of the mean intensity of BODIPY FL-ATP with time. By fitting with single exponential decay, we obtain the bleaching rate constant *k*_2_ = 0.22 ±0.01 s^−1^. **(C,D)** Histograms of *t*_*off*_ and *t*_*on*_ event durations. By fitting with distribution shown in Equation (1) and (2), we obtain the association rate constant *k*_1_ = 1.5 ± 0.2 μM^−1^ s^−1^ and the dissociation rate constant *k*_−1_ = 6.9 ± 0.7 s^−1^.

A typical fluorescent intensity trajectory is shown in Figure 9A. BODIPY FL labeled ATP kept binding and dissociating with AK consecutively. Here, *t*_*off*_, the unbound time, represent the probability of an ATP association event. The dissociation distribution is:

(1) $$\begin{array}{r} P_{on}\left(t\right)=\left[ATP\right]k_{1}e^{-\left[ATP\right]k_{1}t} \end{array}$$

Here, [*ATP*] = 10 nM. The bound time, *t*_*on*_, was affected by the bleaching rate constant *k*_2_ of BODIPY FL and the dissociation rate constant *k*_−1_. The binding distribution (for details, please see Supplementary Information) is:

(2) $$\begin{array}{r} P_{off}\left(t\right)=\left(k_{2}+k_{-1}+k_{2}k_{-1}\right)e^{-\left(k_{2}+k_{-1}\right)t} \end{array}$$

The bleaching rate constant *k*_2_, can be measured independently by observing BODIPY FL-ATP on TIRFM alone. Its distribution function can be written as:

(3) $$\begin{array}{r} P_{b}\left(t\right)=k_{2}e^{-k_{2}t} \end{array}$$

The histograms of *t*_*on*_, *t*_*off*_, *t*_*b*_, and their fitting results were shown in Figures 9B–D. The fitted bleaching rate constant *k*_2_, association rate constant *k*_1_ and dissociation rate constant *k*_−1_ are:

(4) $$\begin{array}{r} k_{2}=0.22\pm 0.01\;{\text{s}}^{-1} \end{array}$$

(5) $$\begin{array}{r} k_{1}=1.5\pm 0.2\;\mu {\text{M}}^{-1}{\text{s}}^{-1} \end{array}$$

(6) $$\begin{array}{r} k_{-1}=6.9\pm 0.7\;{\text{s}}^{-1} \end{array}$$

With the fitted *k*_1_ and *k*_−1_, we can further acquire the dissociation constant $K_{D}=\frac{k_{-1}}{k_{1}}=4.6\pm 1.1\;\mu$M. For *in vitro* experiments, a portion of the protein specimens may lose their activity during purification and observation. Since our method only report active events, those inactive proteins are naturally screened out as a result. This leads to a smaller dissociation constant compared with bulk methods, such as nuclear magnetic resonance (Reinstein et al., 1990).

### Discussion

#### Advantages of single-molecule techniques

Single-molecule fluorescence methods have emerged as powerful tools for studying protein dynamics and reaction kinetics (Joo et al., 2008; Ryu, 2013). By detecting individual biological molecules, single-molecule fluorescence methods not only provide ensemble-averaged values of observables but also reveal information on a molecule's internal distribution. This unique advantage allows single-molecule fluorescence methods to play a crucial role in the investigation of protein kinetics and dynamics. Furthermore, these methods have obvious advantages over traditional biology methods for nonequilibrium systems. Elf et al. (2007) used one such method to directly observe the specific binding of a lac repressor to a chromosomal lac operator in living cells and characterize the kinetics of association and dissociation of the repressor in response to signaling molecules. In combination with reflected light-sheet microscopy, a single-molecule fluorescence method was employed by Gebhardt et al. (2013) to observe the binding of fluorescent-protein-labeled transcription factors on DNA in living mammalian cells. Knight et al. (2015) directly visualized DNA interrogation by Cas9 and observed its off-target binding events in mammalian cells through single-particle tracking. These results demonstrated the advantages of single-molecule methods in life science research.

#### Potential use and challenges of *in Vivo* single-molecule assays

*In vitro* single-molecule methods can be an alternative method of identifying the different features of an entire signaling pathway in living cells. However, some differences exist between *in vitro* and *in vivo* environments. For example, pH and ion concentration are generally heterogeneous *in vivo*, and the cytoplasm has various proteins relevant or irrelevant to the research targets. Therefore, *in vivo* single-molecule methods must be explored.

Thick plant cell walls limit the wide-field imaging of membrane receptors on the cells. Single-molecule assays can be employed through the biochemical removal of the cell wall to obtain protoplasts from the plant cells for observation (Zhang et al., 2011). A new technology, referred to as variable-angle TIRFM (VA-TIRFM), was developed in 2011 (Wan et al., 2011). An evanescent wave is produced inside the cytosol above a cell wall/cytosol interface when the excitation laser has an appropriate incidence angle. Using this constant-depth field, the TIRF image of targets on the plasma membrane can be captured easily. Wang et al. (2015) explored the spatiotemporal dynamics of the BR receptor of *Arabidopsis* BRI1 in live cells using VA-TIRFM.

VA-TIRFM is not suitable for studying downstream signal transduction pathways with a single-molecule resolution, however, because its penetration depth is only 100–200 nm, which is substantially less than cell sizes, which are several micrometers. Epifluorescence microscopy or confocal microscopy can be used instead of VA-TIRFM (Moerner and Fromm, 2003). Nevertheless, autofluorescence from different cellular components—the chloroplasts in plant cells, for example—dramatically increases the background noise and leads to a low SNR and poor image quality (Kodama, 2016). Therefore, fluorophores with extremely high brightness are urgently required for the further development of *in vivo* single-molecule assays.

#### Perspectives of applying single-molecule assays to study plant hormone signal transduction systems

Although single-molecule methods are usually adopted in studies on protein dynamics and reaction kinetics, they are not commonly used to investigate plant hormone signal transduction pathways. Genetic and biochemical techniques have demonstrated remarkable advantages in identifying the key components of such pathways and illustrating their biological functions. However, single-molecule methods are more suitable than traditional methods for obtaining temporal and detailed molecular information about the components. In the ABA cascade, although a fully connected signal pathway has been elucidated using genetic and biochemical approaches, the rate constants between ABA receptor, phosphatase, and their substrates remain unknown. Moreover, the process through which proteins with several substrates, such as MAPK and BIN2, select their substrate in different cell environments remains unclear. Furthermore, it is unknown if the phosphorylation ability of such proteins changes depending on the air or ion concentration and then regulates different hormone signal transduction pathways. These questions are not easily addressed using conventional techniques. Moreover, for transmembrane receptors, such as BRI1 in the BR signaling pathway, the exact conformational changes occurring after their association with BR and that lead to signal transfer into cells are yet to be elucidated. All these dynamic events and detailed reaction mechanisms are difficult to decipher using conventional techniques, necessitating the development of alternative modern technologies.

With advantages such as a well-controlled interaction environment and molecule-by-molecule analysis, single-molecule fluorescence methods can address the aforementioned questions and be powerful tools for studying plant hormone signal transduction pathways. Their potential applications can be increased if they are employed in combination with other modern technologies. For example, by using lipid vesicles to mimic a membrane, the dynamics or conformational changes in membrane receptors before and after ligand binding can be investigated (Boukobza et al., 2001). By combining a single-molecule fluorescence method with optical tweezers, short DNA fragments can be fixed between two beads (Ashkin et al., 1986; Wang et al., 1997). Furthermore, the mechanism through which transcription factors distinguish target sequences or cooperate with each other can be observed directly. By labeling two sites on a receptor or a receptor and its ligand, nanoscale conformational changes as well as ligand binding events can be identified through smFRET. Therefore, the dynamics of macromolecular machinery can be revealed. In summary, single-molecule fluorescent methods open a new avenue for plant signal transduction pathway explorations.

## Conclusion

We have introduced novel TIRFM-based single-molecule fluorescence methods for studying plant hormone signal transduction pathways. Unlike traditional biology methods, these methods focus on the detailed molecular mechanisms of the signaling pathway under investigation. During the preparation step involving protein immobilization on a slide, labeling methods and labeling fluorophores should be carefully determined, particularly while the dye-labeling sites of the protein are being selected. Before microscopic observation, slides should be thoroughly cleaned and passivated. This paper has introduced and validated single-molecule methods combined with monomer regulation, air-controlled flow cell construction, and CoSMoS to study different protein–protein interaction systems. For monomer regulation, divalent streptavidins were used to identify accurate stoichiometry during interactions. For studying signal transduction modulated by gas molecules, a flow cell was constructed to provide airtight conditions and maintain dissolved gas concentration consistency or even change the concentration with time. CoSMoS can be used to study interactions among two or more proteins. By combining CoSMoS with a flow cell model, the protein interaction order, stoichiometry, and molecular mechanisms of two or more fluorophore-labeled proteins can be directly observed simultaneously. On the basis of the observed system, an appropriate model can be constructed to identify the key time constants and conduct the quantitative assessment and dynamic analysis of signal transduction pathways.

## Author contributions

SS and YT designed the experiment. SS, JC, and CM carried out the experiments. SS, CM, JC, and YT wrote the paper.

### Conflict of interest statement

The authors declare that the research was conducted in the absence of any commercial or financial relationships that could be construed as a potential conflict of interest. The reviewer YF and handling Editor declared their shared affiliation.
