# Supplementary material for: Single-Molecule Fluorescence Methods to Study Plant Hormone Signal Transduction Pathways
Source: Front Plant Sci. 2017 Nov 2;8:1888. doi: 10.3389/fpls.2017.01888 (PMC5673658; doi:10.3389/fpls.2017.01888)
Supplement: Supplementary file 1 [file Presentation1.PDF]

Supplementary information：

Derivation of the 'on' probability $P_{on}(t)$ distribution of enzyme binding with one substrate:

For the kinetic model

$$\boldsymbol{E + L \underset{k_{-1}}{\overset{k_1}{\rightleftharpoons}} E \cdot L}$$

$$\downarrow \boldsymbol{k_2}$$

$$\boldsymbol{E + L^b \underset{k_{-1}}{\overset{k_1}{\rightleftharpoons}} E \cdot L^b}$$

$\boldsymbol{E}$, $\boldsymbol{L}$, and $\boldsymbol{L}^b$ represent enzyme, ligand with fluorescent probe, and ligand with bleached probe, respectively. On the intensity-time trajectories, the time durations of low intensities are defined as t_{off}; durations of high intensities are defined as t_{on}. The ending of each t_{on} symbolizes a dissociation event or probe bleaching. Therefore, the 'on' duration t_{on} was affected by the bleaching rate constant k_2 of ligand fluorescent probe and the dissociation rate constant k_{-1}. Their normalized probability distributions are:

$$\boldsymbol{P_b(t) = k_2 e^{-k_2 t}}$$

$$\boldsymbol{P_{dis}(t) = k_{-1} e^{-k_{-1} t}}$$

We cannot distinguish a dissociation event from ligand probe bleaching. Therefore, the probability distribution $\boldsymbol{P_{off}(t)}$ records whichever occurs first or when both events coincide.

If bleaching occurs first, the distribution can be written as:

$$\boldsymbol{P_{b-first}(t) = k_2 e^{-k_2 t}\left(1 - \int_0^t k_{-1} e^{-k_{-1}\tau}\, d\tau\right) = k_2 e^{-(k_2 + k_{-1})t}}$$

Similarly, if dissociation occurs first, the distribution can be written as:

$$\boldsymbol{P_{dis-first}(t) = k_{-1} e^{-k_{-1} t}\left(1 - \int_0^t k_2 e^{-k_2\tau}\, d\tau\right) = k_{-1} e^{-(k_2 + k_{-1})t}}$$

If bleaching and dissociation occur simultaneously,

$$\boldsymbol{P_{sim}(t) = k_{-1} e^{-k_{-1} t} * k_2 e^{-k_2 t} = k_2 k_{-1} e^{-(k_2 + k_{-1})t}}$$

Therefore, the 'on' distribution can be written as:

$$\begin{aligned} \boldsymbol{P_{on}(t)} &= \boldsymbol{P_{b-first}(t) + P_{dis-first}(t) + P_{sim}(t)} \\ &= \boldsymbol{k_2 e^{-(k_2+k_{-1})t} + k_{-1} e^{-(k_2+k_{-1})t} + k_2 k_{-1} e^{-(k_2+k_{-1})t}} \\ &= \boldsymbol{(k_2 + k_{-1} + k_2 k_{-1}) e^{-(k_2+k_{-1})t}} \end{aligned}$$
